# Quality Evaluation of Taxilli Herba from Different Hosts Based on Simultaneous Determination of Multiple Bioactive Constituents Combined with Multivariate Statistical Analysis

**DOI:** 10.3390/molecules26247490

**Published:** 2021-12-10

**Authors:** Nan Wu, Li Li, Zhi-Chen Cai, Jia-Huan Yuan, Wen-Xin Wang, Sheng-Xin Yin, Sheng-Jin Liu, Li-Fang Wei, Yu-Qi Mei, Cui-Hua Chen, Xun-Hong Liu, Li-Si Zou, Jie Li

**Affiliations:** 1 College of Pharmacy, Nanjing University of Chinese Medicine, Nanjing 210023, China; wunan7272@163.com (N.W.); caizhichen2008@126.com (Z.-C.C.); 20200655@njucm.edu.cn (J.-H.Y.); wangwenxin66666666@163.com (W.-X.W.); yinshengxin723@163.com (S.-X.Y.); weilifangquiet@163.com (L.-F.W.); 18260028173@163.com (Y.-Q.M.); cuihuachen2013@163.com (C.-H.C.); zlstcm@126.com (L.-S.Z.); lijieqaz@126.com (J.L.); 2 College of Pharmacy, Guangxi University of Chinese Medicine, Nanning 530220, China; lil2014@gxtcmu.edu.cn

**Keywords:** Taxilli Herba, UFLC-QTRAP-MS/MS, multiple active constituents, simultaneous determination, multivariate statistical analysis

## Abstract

Taxilli Herba (TAXH) is an important traditional Chinese medicine with a long history, dating from the Eastern Han Dynasty to the present times. However, the active constituents in it that parasitize different hosts vary, affecting its clinical efficacy. Given the complexity of the host origins, evaluating the quality of TAXH is critical to ensure the safety and effectiveness of clinical medication. In the present study, a quantitative method based on ultra-fast liquid chromatography tandem triple quadrupole mass spectrometry (UFLC-QTRAP-MS/MS) was established, which simultaneously determined the content of 33 active constituents, including 12 flavonoids, 4 organic acids, 12 amino acids, and 5 nucleosides in 45 samples. Orthogonal partial least squares discriminant analysis (OPLS-DA) was employed to classify and distinguish between TAXH and its adulterants, Tolypanthi Herba (TOLH). A hierarchical clustering analysis (HCA) was conducted combined with a heatmap to visually observe the distribution regularity of 33 constituents in each sample. Furthermore, gray relational analysis (GRA) was applied to evaluate the quality of samples to get the optimal host. The results demonstrated that TAXH excelled TOLH in quality as a whole. The quality of TAXH parasitizing *Morus alba* was also better, while those that were parasitic on *Cinnamomum camphora* and *Glyptostrobus pensilis* had relatively poor quality. This study may provide comprehensive information that is necessary for quality control and supply a scientific basis for further exploring the quality formation mechanism of TAXH.

## 1. Introduction

*Taxillus chinensis* (DC.) Danser is an evergreen shrub of the family loranthaceae—of which the primary medicinal parts are the stems, branches, and leaves—named Taxilli Herba (TAXH). Its harvest occurs from winter to spring, after which thick stems are removed, cut into sections, dried, or dried after steaming before finally becoming TAXH [1]. It is traditional Chinese medicine (TCM), which is widely used in TCM clinical practice and Chinese patent medicine production. Additionally, it is used for making tea, with the objective of achieving good health in daily life. The earliest record of TAXH can be traced back to the Chinese Han Dynasty in Shennong’s Herbal Classic of Materia Medica, with the traditional function of dispelling wind-damp, strengthening bones and muscles, and preventing miscarriage, which is divided into tonic, in top grade [2]. Previous phytochemical studies have revealed that TAXH is rich in a variety of important phytochemicals such as flavonoids, organic acids, volatile oil, phospholipids, vitamins, and trace elements [3,4,5,6,7,8,9,10]. Modern pharmacology research indicates that TAXH is not only able to inhibit tumor growth and induce cell apoptosis [11,12], decrease the blood fat [13], lower blood pressure [14], and fight off viruses [15], but it also possesses the obvious effect of anti-inflammation and analgesia [16]. More than that, it has an antioxidant effect and neuroprotective activity [17,18]. These excellent pharmacological benefits are attributed to the bioactive constituents in this Chinese medical herb.

Prior research on the active constituents of TAXH, mostly focused on the quantitative analysis of either total flavonoids or a single active constituent [19,20,21] and on the synergistic action of various constituents, was supposed to be responsible for the therapeutic effects of TCM. Therefore, a systematic approach to the simultaneous determination of multiple active constituents is required for a comprehensive assessment of the quality of TAXH. Firstly, flavonoids and organic acids are the main efficacious constituents of TAXH, which contribute remarkably to its efficacy in anti-cancer, anti-inflammatory, vascular protection, and antioxidant activities [22,23,24,25,26,27,28]. Secondly, amino acids and nucleosides also possess various biological activities [29]. At present, no quantitative analysis of amino acid and nucleoside constituents of TAXH has been reported. We selected a total of 12 amino acids and 5 nucleosides for quantitative analysis based on essential amino acids and medicinal amino acids, excluding those not detected in the pre-experiment and those with very low response values. Ultimately, a total of 33 constituents were selected for analysis, which generally covers the chemical structure types of the main active constituents and important primary metabolites in TAXH; the variation of their contents can depict the quality of the herbs to a large extent. Simultaneously, in previous studies on TAXH, the commonly used analytical method for it was high-performance liquid chromatography (HPLC) [21], which is time-consuming [30] and has difficulties in effectively separating compounds with similar polarities [31]. UFLC-QTRAP-MS/MS effectively resolves the problem of difficult separation of complex systems in HPLC [32]. It has also been widely used for the separation and analysis of metabolites in original herbal medicine [33,34]. Therefore, the UFLC-QTRAP-MS/MS technology was adopted for separation or detection in this study.

According to statistics, there are more than 20 species used as TAXH, accounting for more than 80% of the number of species in the Loranthaceae family in Guangxi [35]. The constituents of most parasites and the differences with TAXH have been studied before, while the quality of Tolypanthi Herba (TOLH) has rarely been investigated. Although both TAXH and TOLH are of the Loranthaceae family and have certain morphological similarities, they have certain differences in constituents and efficacy and should be distinguished for clinical use. Therefore, an effective method must be adopted to differentiate between the two species in order to ensure the quality of TAXH.

The purpose of this experiment is to evaluate the quality of Taxilli Herba from different hosts through the simultaneous determination of multiple active constituents combined with a multivariate statistical analysis. Accordingly, in this study, ultra-fast liquid chromatography coupled with triple quadrupole–linear ion trap tandem mass spectrometry (UFLC–QTRAP–MS/MS) was performed to simultaneously determine the contents of 33 bioactive constituents, including 12 flavonoids, 4 organic acids, 5 nucleotides, and 12 kinds of amino acids in the samples. Next, orthogonal partial least squares discriminant analysis (OPLS-DA) was introduced to distinguish between TAXH and TOLH, and a clustering heat map was drawn to cluster the different hosts of TAXH and TOLH by hierarchical cluster analysis (HCA). Finally, grey relational analysis (GRA) was performed to evaluate the quality of the samples based on the content of the 33 analytes. The present research lays a valuable foundation for in-depth investigations on the overall quality of TAXH and the determination of the optimal hosts.

## 2. Results

### 2.1. Optimization of Sample Preparation

Quercetin is an essential efficacy constituent of TAXH, which exists mainly in the form of quercitrin and only occasionally in its free form. Hence, the extraction rate of quercitrin was used as the response value in the selection of the extraction process. To optimize the extraction procedure, certain variables that could impact the extraction efficiency were chosen. First, the effects of the volume fractions of solvent, the liquid-to-material ratio, and the extraction time for the yields of quercitrin were evaluated individually through single-factor experiments (Figure S1). The three-parameter settings were as follows: volume fractions of solvent (40% methanol, 50% methanol, 60% methanol, 70% methanol, 80% methanol, 90% methanol), liquid-to-material ratio (10:1 mL/g, 20:1 mL/g, 30:1 mL/g, 40:1 mL/g, 50:1 mL/g, 60:1 mL/g), and extraction time (20 min, 30 min, 40 min, 50 min, 60 min, 70 min).

In the next step, the Box–Behnken design (BBD) along with response surface methodology (RSM) were employed for further optimization of the extraction condition. The levels and codes of extraction variables used in the BBD are shown in Table S1. Based on the results of 17 test points tested by BBD in random order, the experimental design and response value are shown in Table S2. Combined with multiple regression analysis, a relationship between the response and the variables was obtained and expressed by the following second-order polynomial equation:

(1) $$\begin{array}{l} Y=3.93+0.074\times X_{1}+0.091\times X_{2}+0.035\times X_{3}-0.36\times X_{1}\\ \;\times X_{2}-0.085\times X_{1}\times X_{3}-3.000E-003\times X_{2}\times X_{3}-0.68\\ \;\times X_{1}^{2}-0.67\times X_{2}^{2}-0.092\times X_{3}^{2} \end{array}$$

where *Y* (mg/g) is the response value of the quercitrin content, *X*_1_, *X*_2_, *X*_3_ are the volume fractions of solvent, liquid-to-material ratio, and extraction time, respectively. 

The analysis of variance (ANOVA) of the experimental results of the BBD are shown in Table S3. The *p* and *F* values of the model were <0.0001 and 33.39, respectively, indicating that the regression model was highly significant. Meanwhile, the “lack-of-fit” *p*-value (0.7715) and *F*-value (0.38) suggested that the “lack-of-fit” was not significant relative to the pure error. As presented in Figure S2, the 3D response plot shows the combined effects of methanol concentration, extraction time, and the liquid-to-material ratio. It can be deduced that the optimal extraction conditions are the following: 70% methanol, a liquid-to-material ratio of 30.60:1, and 31.79 min for extraction. Under these optimized conditions, a parallel test in triplicate was used to verify the reliability of the model. The experimental results demonstrated that the average extraction rate was 3.77 mg/g, which is 0.17% different from the predicted value of 3.94 mg/g. It means that the optimized extraction conditions were reliable, and that they could be applied further for the extraction of TAXH.

### 2.2. Optimization of UFLC Conditions

Different chromatographic columns were investigated, including the XBridge^®^C18 column (4.6 mm × 100 mm, 3.5 µm) and the SynergiTM Hydro-RP 100 Å column (2.0 mm × 100 mm, 2.5 µm). Based on the results of UFLC, the former was eventually selected considering both the peak shape and the separation of the target compounds. Moreover, three kinds of mobile phase systems (water/methanol, water/alcohol, water/acetonitrile, water (containing 0.1% formic acid)/methanol) were compared. The column temperature (25 °C, 30 °C, 35 °C) and flow rate (0.3 mL/min, 0.5 mL/min, 0.8 mL/min) were optimized further. A gradient elution using 0.1% formic acid as Eluent A and methanol as Eluent B, at a flow rate of 0.5 mL/min under the column temperature of 30 °C achieved the desired separation.

### 2.3. Optimization of MS Conditions

**Figure 1:**
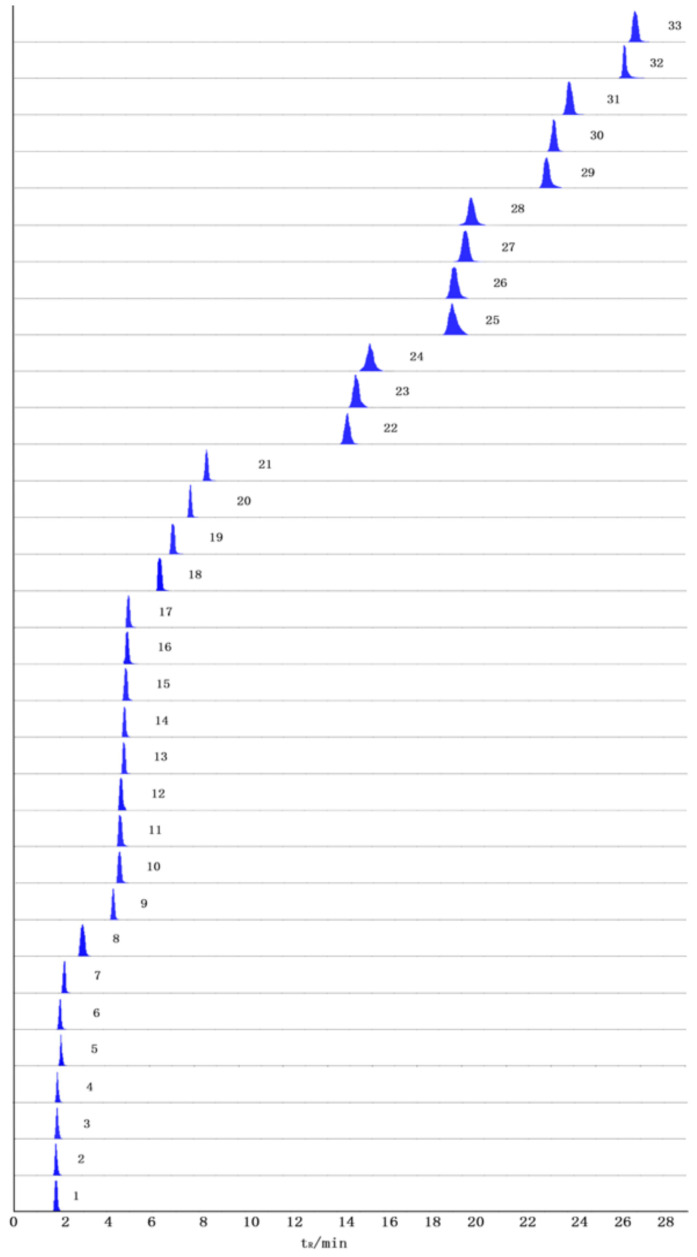
Representative extract ion chromatograms (XIC) of multi-reaction monitoring (MRM) chromatograms of 33 investigated constituents in the samples.

The individual solutions of all standard compounds (about 100 ng/mL) were examined with the electrospray ionization (ESI) source in both positive and negative ion modes to optimize instrumental parameters for MS/MS detection. Through trial and error, we reached the same conclusion that the overwhelming majority of flavonoids and organic acids have sensitivity and intensity in the negative ion mode, while avicularin, amino acids, and nucleosides have a relatively strong response in the positive ion mode. For this reason, the ESI^+^ and ESI^−^ modes were simultaneously performed in this experiment. The multiple reaction monitoring (MRM) chromatograms of 33 compounds are shown in Figure 1. In addition, the optimum parameters, including the retention time (t_R_), precursor and product ions, De-clustered Voltage (DP), and collision energy (CE) of 33 compounds are summarized in Table 1. As shown, Leucine-Isoleucine, Hyperin-Isoquercetin, and Quercetin-3-*O*-(6″-galloyl)-*β*-D-galactopyranoside, Quercetin-3-*O*-(6″-galloyl)-*β*-D-glucopyranoside were isomers with the same precursor ion–product ion pairs, therefore, every single standard solution was injected into UFLC-QTRAP-MS/MS, and the compound was determined with the aid of t_R_.

### 2.4. Method Validation

The proposed UFLC-QTRAP-MS/MS method was validated by determining the linearity, limits of detection and quantification (LOD and LOQ), precision, repeatability, stability, and recovery rate. The standard curves were drawn by plotting the peak area (Y) versus the corresponding concentration (X, ng/mL), which exhibited good linearity with appropriate correlation coefficients (r^2^ > 0.9990). The LODs and LOQs for all compounds were 0.182–40.597 ng/mL and 0.608–135.324 ng/mL, correspondingly. Precision was studied as intra-day and inter-day precision. The relative standard deviations (RSDs) were calculated as 1.07–3.34% and 0.97–3.86%, illustrating the proposed method’s high precision. The repeatability was assessed by analyzing six independently prepared samples using the same method, and one of the sample solutions was analyzed at 0, 2, 4, 8, 12, and 24 h, correspondingly, to evaluate the stability. Our results indicate that this method had good repeatability (RSD: 1.38–4.67%) and stability (RSD: 0.86–4.58%). The analytical results in Table 1 show that the spiked recoveries were in the range of 98.03–101.32%, with RSD ranges of 1.02–3.40%, confirming the accuracy of the presented method. The slope ratio values of the matrix curve to the pure solution curve are between 0.91 and 1.04, demonstrating that the matrix effects on the ionization of analytes were so small as to be negligible for this assay. The detailed results are presented in Table 2. 

### 2.5. Determination of Samples

Sample information is listed in Table 3. The UFLC–QTRAP–MS/MS methodology established in this paper has been applied to the comprehensive quality evaluation. The quantitative results of 33 active constituents in these samples are summarized in Table S4. Analyses of all samples were performed in triplicate for the average. All four classes of constituents were detected in 45 samples, and all samples contained high levels of flavonoids. These findings accord with earlier documents, suggesting that the constituents in TAXH are host-dependent in degree, but not in kind. Significant differences were shown in the contents of bioactive constituents between TAXH and TOLH. More specifically, what is most noticeable is that the flavonoid content in TAXH ranged from 2076.27 μg/g to 74,381.34 μg/g, while the content range of TOLH was 2877.38–5753.95 μg/g. It can be clearly observed from Figure S3 that except for organic acids, the content of the other three kinds of constituents was higher in TAXH as a whole. Additionally, the large variation in the content of active constituents in TAXH from different hosts illustrates that it is crucial to evaluate their quality.

### 2.6. OPLS-DA of Samples

**Figure 2:**
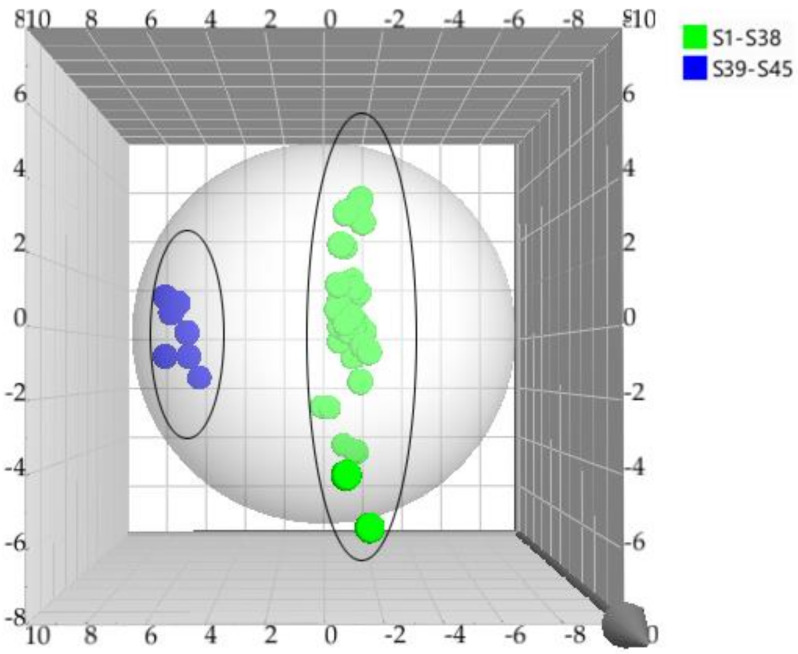
The OPLS–DA model for the classification of TAXH and TOLH based on the content of 33 constituents.

**Figure 3:**
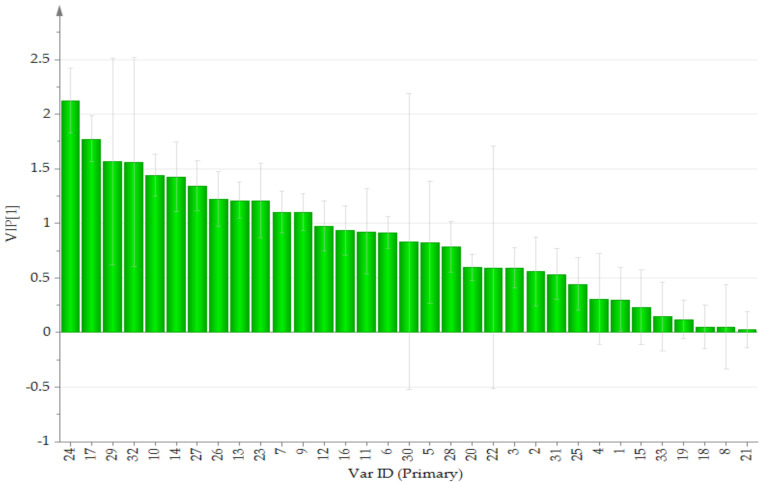
VIP for classification of TAXH and TOLH. The VarID is the same as that in Table 1.

Initially, principal component analysis (PCA) was performed to distinguish and assess the quality of TAXH and TOLH. However, it could not be observed that the determined samples were completely divided into two clusters. Orthogonal partial least squares discriminant analysis (OPLS-DA), a supervised latent structures discriminant analysis method, which can maximize the difference between groups and minimize the separation between intra-group was applied. To better classify samples and identify the differential metabolites, the data were subjected to multivariate statistical analysis, and OPLS-DA was performed. The OPLS-DA score plot is presented in Figure 2. TAXH and TOLH were separated into two groups, thereby indicating the remarkable differences of chemical constituents between them. In general, R2 describes how well the model is fitted. Q2 describes how well the X could predict the Y. In this comparison, the statistical parameters of OPLS-DA R2X (cum), R2Y (cum), and Q2(cum) were 0.969, 0878, and 0.843, respectively, indicating the good repeatability and predictability of the model. The variable importance of projection (VIP) is the vector that summarizes the total importance of the variable in explaining the model. If a variable has a VIP > 1.0, it indicates that the variable strongly contributes to the classification of those samples. As shown in Figure 3, eight constituents were found to play leading roles in the cluster based on the VIP values, including quercetin-3-*O*-*β*-D-glucuronide, isoquercitrin, catechin, hyperin, proline, quercetin, quercetrin, and glutamic acid.

### 2.7. HCA of Samples

**Figure 4:**
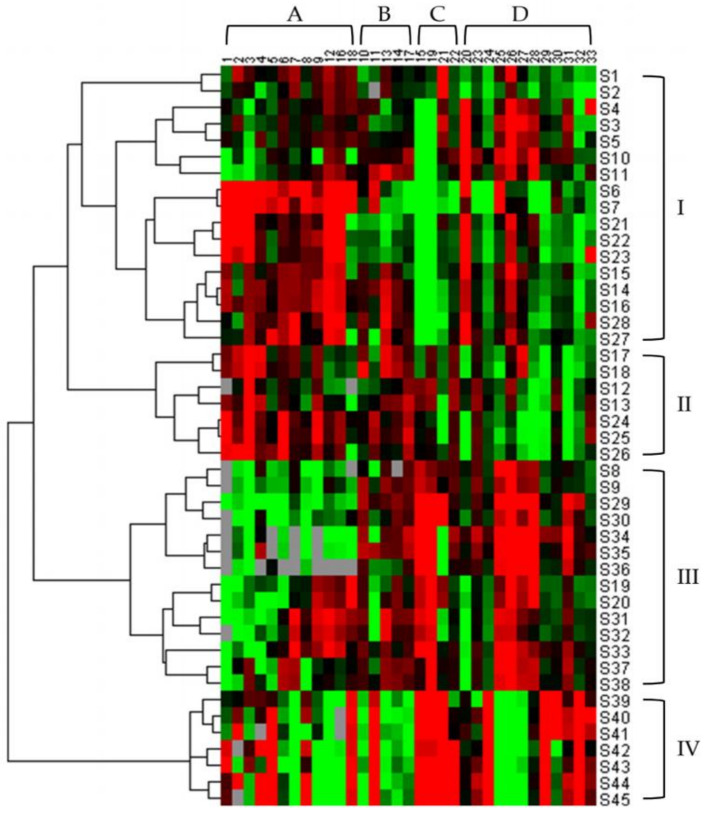
Hierarchical clustering analysis of TAXH from different hosts and TOLH. The heatmap shows the content difference of 33 constituents. The green color represents the decreasing trend, the red represents an increasing trend. A total of 45 samples were divided into 4 categories, which were Category I, Category II, Category III, and Category IV. (Amino acids (**A**), Nucleosides (**B**), Organic acids (**C**), Flavonoids (**D**)).

HCA is one of the most commonly used methods of multifactorial analysis, which not only visualizes complex data but also provides a way to assess similarity among samples [36]. Combined with heat maps, it makes the level of active constituents and the relationship among samples more obvious. To visually classify samples and observe the differences of 33 constituents in samples, HCA was applied in this study. Figure 4 shows the resulting dendrogram. Any of the squares in the diagram indicates the amount of a constituent in a given sample, with the color ranging from green to red, representing low to high levels. As can be seen from the diagram, owing to the similarity in the content of constituents, the TAXH with the hosts of *Morus alba*, *Amygdalus persica*, *Clausena lansium,* and *Diospyros kaki* clustered into one group, Category I; those parasitic on *Clausena excavata*, *Cinnamomum camphora,* and *Glyptostrobus pensilis* were grouped into Category II; others that grew on the remaining hosts fell into Category III, and ultimately, these three categories were clustered into one category in order to be distinguished from the Category IV of TOLH.

It is also evident from the graph that contents of all types of compounds are relatively high in the samples from Category I; flavonoids are less abundant in the Category II samples, while amino acids and nucleosides are plentiful; samples in Category III contain high amounts of both flavonoids and organic acids; organic acids are comparatively high in the Category IV samples. From the results, it can be inferred that the content of bioactive constituents in TAXH that are parasitizing different hosts varies substantially.

### 2.8. GRA of Samples

Gray correlation analysis, pioneered by Deng Julong in 1982 [37], is a methodology for quantitative comparisons. It uses the correlation degree between the referring series and the comparing series to evaluate the proposed schemes [38]. Moreover, it can reflect the comprehensive quality of samples in a more authentic and overall way. The steps for calculating the correlation degree can be summarized as follows:

(1) Normalized processing of the original data

First, the original index value should be normalized as the value belonging to [0, 1] in order to make sure of the evaluation accuracy [39]. For simplicity, we assumed that there are n samples and m evaluation indices for each sample. These composed sequences: {*X_ik_*} (i = 1, 2, 3…*n*; k = 1, 2, 3…*m*; In this experiment, *n* = 45, *m* = 33). The formula is as follows:

(2) $$Y_{ik}=\frac{X_{ik}}{X_{k}}$$

where *X_ik_* is the raw data and *Y_ik_* is the processed data. *X_k_* is the average value of n samples in the kth index.

(2) Calculation of grey relational coefficient

It is necessary to consider the optimal reference sequence and the worst when statistical evaluations are performed using the gray relational analysis. The optimal reference sequence is {*X_sk_*} (k = 1, 2, 3…*m*); the worst is {*X_tk_*} (k = 1, 2, 3…*m*). Their relational coefficient could be calculated separately by Formulas (3) and (4).

(3) $$\xi_{k\left(s\right)}=\frac{\Delta \min+\rho \Delta \max}{|Y_{ik}-Y_{sk}|+\rho \Delta \max}$$

where $\Delta \min=\min|Y_{ik}-Y_{sk}|,\Delta \max=\max|Y_{ik}-Y_{sk}|$ (i = 1, 2, 3…*n*; k = 1, 2, 3…*m*)

(4) $$\xi_{k\left(t\right)}=\frac{\Delta^{\prime }\min+\rho \Delta^{\prime }\max}{|Y_{ik}-Y_{tk}|+\rho \;\Delta \prime \max}$$

where $\Delta^{\prime }\min=\min|Y_{ik}-Y_{tk}|,\Delta^{\prime }\max=\max|Y_{ik}-Y_{tk}|$(i = 1, 2, 3…*n*; k = 1, 2, 3…*m*), $\rho \in \left[0,\;1\right]$ is introduced as the resolution coefficient to reduce the influence of the extreme value, which is usually 0.5.

(3) Calculation of relational grade

The relational grade of the optimal reference sequence and the worst could be calculated by Formulas (5) and (6), respectively.

(5) $$r_{i\left(s\right)}=\frac{1}{m}\overset{m}{\underset{k=1}{\sum }}\xi_{k\left(s\right)}^{i}$$

(6) $$r_{i\left(t\right)}=\frac{1}{m}\overset{m}{\underset{k=1}{\sum }}\xi_{k\left(t\right)}^{i}$$

(4) Calculation of the relative relational grade is as follows:

(7) $$r_{i}=\frac{r_{i\left(s\right)}}{r_{i\left(s\right)}+r_{i\left(t\right)}}$$

The grey comprehensive evaluation values (*r_i_*) and quality-rankings are listed in Table 4. It is clear that the overall quality of TAXH is superior to that of TOLH from this point of view. More than that, there is a great difference in quality between TAXH from different hosts. This is also true for TOLH. The TAXH from *Morus alba*, *Amygdalus persica*, and *Tabernaemontana divaricata* had better quality as compared to those from other hosts. Within them, the TAXH that lived on *Morus alba* was of the finest quality. Relatively speaking, the quality of TAXH whose host was *Cinnamomum camphora* and *Glyptostrobus pensilis* was poorer. These results coincide with the HCA results described above. In the meantime, the difference values of *r_i_* showed a large variation, with a maximum value of 33.8%, which could well distinguish the quality of the samples. As can be seen from the table, the quality of S8 and S7 is much better than that of S1, S2, and S3 when their hosts are the same. The reason for this may be that geographical location and harvesting time have some influence on quality. In summary, the quality of TAXH can be successfully assessed by GRA based on the content of their multiple constituents.

## 3. Discussion

Based on previous studies, we predicted that the quality of TAXH living on the different hosts would vary, and that the best quality would be the TAXH parasitic on *Morus alba*; we also surmised that TAXH would significantly differ from TOLH. In the present study, the quantitative analysis of 33 constituents in 45 samples revealed the TAXH with *Morus alba* as the host is of the best quality, and the quality of TAXH parasitizing 13 different hosts were significantly different. These results were not only consistent with prior research, but also with those recorded in ancient books [40,41]. Meanwhile, we also found that the TAXH living on *Cinnamomum camphora* was of the worst quality. In addition, it was also found that TAXH and TOLH could be clearly distinguished; the quality of TOLH was apparently inferior to that of TAXH, and it was not reasonable to use TOLH as a substitute for TAXH. Similarly, among the TOLH samples, those whose host was *Morus alba* had superior quality. All the samples in this study were of the same origin, i.e., Guangxi Province, the genuine producing area of TAXH, which objectively avoided the influence and bias caused by factors of origin. Our findings provide a scientific reference for the study of the quality of TAXH and a theoretical basis for an in-depth investigation of the quality formation mechanism of TAXH. Nevertheless, our study has several limitations: firstly, we failed to cover more hosts, and secondly, the constituents studied were all intrinsic to TAXH, and we did not investigate the constituents that had been transmitted from hosts to TAXH. These may affect the results, and this is the part we need to continue to explore in the future. We will do further research on the reasons for the differences as well. Apropos of investigating how the clinical efficacy is influenced by the content variations of the constituents also remains to be an important task.

## 4. Materials and Methods

### 4.1. Plant Materials

**Figure 5:**
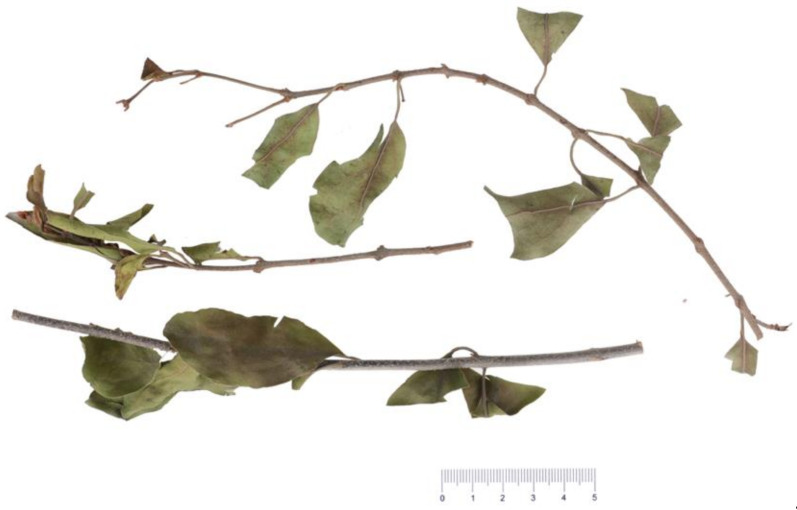
The Taxilli Herba from *Morus alba*.

Forty-five samples of TAXH (S1–S38) and TOLH (S39–S45) were collected from Guangxi Province in China. All the samples were authenticated by Professor Xunhong Liu (Nanjing University of Chinese Medicine, Nanjing, China) and were deposited in the laboratory of Chinese medicine identification, Nanjing University of Chinese Medicine. A picture of the herbs is shown below (Figure 5).

### 4.2. Chemicals and Reagents

The standards of rutin, quercetin, gallic acid were purchased from National Institutes for Food and Drug Control (Beijing, China). Hyperin, auicularin, catechin, quercetin-3-*O*-(6″-galloyl)-*β*-D-galactopyranoside, quercetin-3-*O*-(6″-galloyl)-*β*-D-glucopyranoside, coniferic acid, histidine, arginine, lysine, serine, theronine, glutamic acid, proline, valine, tyrosine, leucine, isoleucine, phenylalanine, 2′-deoxyadenosine, 2′-deoxycytidine, adenosine, inosine, and guanosine were purchased from Yuanye Biotechnology Co., Ltd. (Shanghai, China). Quercetrin was purchased from Chinese National Institute of Control of Pharmaceutical and Biological Products (Beijing, China). Isoquercitrin was purchased from Chengdu Chroma-Biotechnology Co., Ltd. (Chengdu, China). Kaempferol-3,7-bisrhamnoside was purchased from Chengdu Alfa Biotechnology Co., Ltd. (Chengdu, China). Isosakuranetin and quercetin-3-*O*-*β*-D-glucuronide were purchased from Liangwei Biotechnology Co., Ltd. (Nanjing, China). Protocatechuic acid was purchased from Shanghai Ronghe medical-technology Co., Ltd. (Shanghai, China). Chlorogenic acid was purchased from Baoji Chenguang Biotechnology Co., Ltd. (Baoji, China). The purities of the above-mentioned constituents were more than 98%, according to HPLC analysis. Methanol and formic acid of HPLC grade were purchased from Merck (Darmstadt, Germany). Ultrapure water used in all the experiments was prepared using a Milli-Q purifying system (Millipore, Bedford, MA, USA).

### 4.3. Preparation of Standard Solutions

A mixed standard stock solution of 33 standards was prepared by dissolving accurately weighed standards in 70% methanol. The concentration of each standard was as follows: 4.9, 5.8, 17.0, 5.2, 5.1, 30.6, 70.8, 5.1, 5.1, 5.0, 5.3, 5.2, 5.2, 5.1, 5.7, 5.2, 5.5, 5.3, 5.8, 5.8, 97.0, 5.0, 5.0, 5.1, 126.6, 39.5, 5.1, 401.2, 5.0, 5.0, 145.5, 5.0, 50.5 μg/mL.

The mixed reference solution was diluted step by step with 70% methanol to obtain a series of mixed reference solutions at different concentrations for the construction of calibration curves. All the solutions were stored at 4 °C and were filtered through a 0.22 μm membrane before injection into the UFLC system.

### 4.4. Preparation of Sample Solutions

The fresh samples were collected and dried in the oven at 40 °C, after which they were crushed with a pulverizer and sieved through a 50-mesh screen. Then, 0.5 g powder was accurately weighed and placed in a conical flask with a stopper. An exact amount of 15 mL of 70% methanol was added into the conical flask and extracted by ultrasonic extraction for 32 min. After cooling down to room temperature, 70% methanol was added to compensate for the weight loss. After centrifugation (12,000 r/min, 10 min), the supernatant was collected and diluted tenfold, followed by filtering through a 0.22 µm microporous membrane before LC–MS analysis.

### 4.5. Chromatographic and Mass Spectrometric Conditions

The SIL–20A UFLC XR system (Shimadzu Co., Kyoto, Japan) was used for analysis. LC separation was performed on an XBridge^®^C18 column (4.6 mm × 100 mm,3.5 µm) at 30 °C under the condition of gradient elution, with the mobile phase consisting of 0.1% formic acid (A)-MeOH(B). The gradient elution was as follows: 0–5 min, 2–27% A; 5–8 min, 27–31% A; 8–14 min, 31–32% A; 14–17 min, 32–34% A; 17–22 min, 34–40% A; 22–26 min, 40–73% A; 26–29 min, 73–2% A. The injection volume was 2 μL, and the flow rate was 0.5 mL/min.

An API5500 triple quadrupole linear ion trap tandem mass spectrometer (AB SCIEX, Framingham, MA, USA) equipped with an electrospray ionization (ESI) source was used for detection. The operating parameters were as follows: ion source temperature, 550 °C; nebulizer gas (GS1) flow, 55 L/min; auxiliary gas (GS2) flow, 55 L/min; curtain gas (CUR) flow, 40 L/min; spray voltage (IS), 4500 V in the positive mode and −4500 V in the negative mode. Detection of analytes was performed in multiple-reaction mode (MRM).

### 4.6. Validation of the Method

The method was validated with regard to linearity, precision of intra-day and inter-day, repeatability, stability, recovery, and matrix effect.

Serial dilutions of mixed standards were used to establish the standard curves, and the linear regression equation, correlation coefficient, and linear range were calculated. The detection limit (LOD) and quantification limit (LOQ) for 33 constituents were calculated at the signal-to-noise ratio of 3 and 10, respectively. For intra-day precision, the mixed standards solutions were injected for six replicates within one day, while for inter-day precision, the solutions were examined in triplicates for 3 consecutive days. To validate the repeatability, six samples of TAXH were accurately weighed and prepared independently according to the optimal conditions above and then analyzed. The same sample solution was taken and determined at 0, 2, 4, 8, 12, and 24 h, correspondingly, according to the above chromatographic conditions to evaluate the stability. The recovery experiments were used to assess the accuracy of the method; standards at three different concentration levels, including low (80%), median (100%), and high (120%) were added to samples of known content. Each experiment was repeated three times, and the spiked samples were analyzed by UFLC–QTRAP–MS/MS to evaluate the recoveries. The recoveries were calculated by the formulae: recovery (%) = (detected amount − original amount)/spiked amount × 100%. The matrix effect refers to the enhancement or suppression of a chromatography signal by interference or co-eluting compounds in the matrix [42]. It was evaluated using a slope comparison method. In this way, the matrix effect was determined to be the ratio of the slope in a matrix-matched calibration curve to the slope in a solvent standard curve. The slope ratio close to 1.0 indicates that the matrix effect is weaker [43].

### 4.7. Statistical Analysis

After data preprocessing, OPLS-DA was applied to observe the global clustering trends of various groups and to visualize their distribution by using SIMCA-P 13.0 software (Umetrics AB, Umea, Sweden). Cluster analysis was performed and heatmaps were plotted using Cluster 3.0 (De Hoon et al., 2004) and Java TreeView1.2.0 (Alok J. Saldanha, 2004) in order to visualize the classification and content changes of the samples more intuitively. GRA was adopted on the basis of the contents of 33 active constituents to evaluate the quality of the samples of TAXH and TOLH by using Excel for Mac 2019 (Microsoft Corp., Seattle, WA, USA). All the histograms were charted with OriginPro 2021b (OriginLab, Northampton, MA, USA).

## 5. Conclusions

In this study, 33 active constituents were quantified by UFLC–QTRAP–MS/MS in 45 samples of TAXH and TOLH. An OPLS–DA model was developed to discriminate between TAXH and TOLH. Eight constituents (quercetin-3-*O*-*β*-D-glucuronide, isoquercitrin, catechin, hyperin, proline, quercetin, quercetrin, glutamic acid) were finally analyzed as the key constituents in order to distinguish these two medicinal herbs. By means of HCA, not only were TAXH and TOLH divided into two major groups, but TAXH with similar quality were also grouped into one cluster. Furthermore, GRA was conducted to assess the quality of the samples. Based on these, the following conclusions have been drawn: The overall quality of TAXH was better than that of TOLH, and the host was the key factor affecting the quality of TAXH. These findings indicate that simultaneous determination of multiple bioactive constituents combined with multivariate statistical analysis can be used to distinguish the two herbs, TAXH and TOLH, and can assess the quality of TAXH from different hosts. Conclusively, the implemented method shows efficacy and its potential application in the quality control of TAXH and may provide a theoretical basis for the comprehensive evaluation of TAXH.

## Figures and Tables

**Table 1:** Optimized mass spectrometric parameters for MRM of 33 constituents.

No.	Constituents	Formula	TR (min)	MRM Parameters			
				MRM Transitions (*m*/*z*)	DP (V)	CE (eV)	Ion Mode
1	Lysine	C_6_H_14_N_2_O_2_	1.81	147.11/83.91	100	14	ESI+
2	Histidine	C_6_H_9_N_3_O_2_	1.85	156.09/110.03	130	32	ESI+
3	Argnine	C_6_H_14_N_4_O_2_	1.88	175.12/70.02	88	18	ESI+
4	Serine	C_3_H_7_NO_3_	1.96	106.05/59.99	100	8	ESI+
5	Theronine	C_4_H_9_NO_3_	2.04	120.07/74.00	100	2	ESI+
6	Glutamic acid	C_5_H_9_NO_4_	2.07	148.08/83.91	12	14	ESI+
7	Proline	C_5_H_9_NO_2_	2.25	116.07/70.02	68	10	ESI+
8	Valine	C_5_H_11_NO_2_	3.06	118.09/72.06	100	10	ESI+
9	Tyrosine	C_9_H_11_NO_3_	4.38	182.08/136.01	16	16	ESI+
10	Adenosine	C_10_H_13_N_5_O_4_	4.63	268.10/136.10	86	23	ESI+
11	2′-Deoxyadenosine	C_10_H_13_N_5_O_3_	4.7	252.40/136.10	50	18	ESI+
12	Isoleucine	C_6_H_13_NO_2_	4.8	132.20/86.05	64	10	ESI+
13	Inosine	C_10_H_12_N_4_O_5_	4.88	269.00/137.00	46	15	ESI+
14	Guanosine	C_10_H_13_N_5_O_5_	4.89	284.30/152.10	42	16	ESI+
15	Gallic acid	C_7_H_6_O_5_	4.98	169.00/125.00	−33	−13	ESI-
16	Leucine	C_6_H_13_NO_2_	5.04	132.20/86.00	64	10	ESI+
17	2′-Deoxyguanosine	C_10_H_13_N_5_O_4_	5.07	268.00/152.30	39	13	ESI+
18	Phenylalanine	C_9_H_11_NO_2_	6.46	166.10/120.05	100	14	ESI+
19	Protocatechuic acid	C_7_H_6_O_4_	7.07	152.94/109.00	−85	−16	ESI−
20	Catechin	C_15_H_14_O_6_	7.88	289.00/244.80	−180	−20	ESI−
21	Chlorogenic acid	C_16_H_18_O_9_	8.59	353.14/190.90	−35	−20	ESI−
22	Coniferic acid	C_10_H_10_O_4_	14.87	193.00/133.90	−27	−24	ESI−
23	Quercetin-3-*O*-(6″-galloyl)-*β*-D-galactopyranoside	C_28_H_24_O_16_	15.26	615.02/463.05	−180	−38	ESI−
24	Quercetin-3-*O*-(6″-galloyl)-*β*-D-glucopyranoside	C_28_H_24_O_16_	15.9	615.02/463.05	−180	−38	ESI−
25	Quercetin-3-*O*-*β*-D-glucuronide	C_21_H_18_O_13_	19.55	477.09/300.97	−110	−32	ESI−
26	Hyperin	C_21_H_20_O_12_	19.67	462.936/300.00	−155	−36	ESI−
27	Rutin	C_27_H_30_O_16_	20.24	608.945/299.90	−170	−48	ESI−
28	Isoquercitrin	C_21_H_20_O_12_	20.37	462.90/300.00	−155	−36	ESI−
29	Auicularin	C_20_H_18_O_11_	23.9	435.00/303.00	130	15	ESI+
30	Kaempferol-3,7-bisrhamnoside	C_27_H_30_O_14_	24.05	577.13/282.99	−200	−52	ESI−
31	Quercetrin	C_21_H_20_O_11_	24.76	447.00/301.00	−180	−30	ESI−
32	Quercetin	C_15_H_10_O_7_	27.24	301.10/151.00	−62	−28	ESI−
33	Isosakuranetin	C_16_H_14_O_5_	27.76	285.07/164.09	−120	−28	ESI−

**Table 2:** Regression equations, limits of detection (LOD) and limits of quantification (LOQ), precision, repeatability, stability, recovery, and matrix effect of 33 constituents.

No.	Constituents	Regression Equation	r	Liner Range (ng/mL)	LOD (ng/mL)	LOQ (ng/mL)	Precision (RSD, %)		Repeatability (RSD, %)	Stability (RSD, %)	Recovery (%)		Matrix Effect
							Intra-Day (*n* = 6)	Inter-Day (*n* = 9)			Mean	RSD	
									(*n* = 6)	(*n* = 6)			
1	Lysine	Y = 2870X + 29,900	0.9995	19.297–4940	3.938	13.127	2.32	1.42	1.64	1.12	100.30	2.08	0.92
2	Histidine	Y = 1120X – 11,500	0.9998	44.922–2875	8.751	29.170	1.07	2.98	3.47	2.31	100.50	2.04	0.94
3	Argnine	Y = 7830X – 135,000	0.9992	8.301–2125	0.711	2.372	1.24	0.97	1.55	2.45	101.32	1.57	0.91
4	Serine	Y = 1050X + 32,600	0.9997	80.625–5160	14.397	47.991	3.08	1.82	3.95	2.05	99.83	1.02	0.96
5	Theronine	Y = 1430X + 56,100	0.9992	39.531–5060	6.663	22.209	1.41	1.36	2.88	2.40	99.61	3.17	1.02
6	Glutamic acid	Y = 1390X + 85,400	0.9998	59.766–15,300	11.277	37.588	2.35	2.06	1.69	1.65	99.96	1.50	0.99
7	Proline	Y = 5530X + 2,310,000	0.9993	8.647–4427.5	1.540	5.132	2.52	1.42	2.82	2.89	99.77	2.10	0.97
8	Valine	Y = 14,200X – 74,600	0.9998	19.844–2540	4.314	14.380	1.24	1.14	3.66	0.87	100.24	2.32	1.03
9	Tyrosine	Y = 3580X + 244,000	0.9995	20–5120	5.728	19.093	2.85	2.22	4.44	1.30	99.40	3.40	1.04
10	Adenosine	Y =32,700X + 414,000	0.9991	2.432–622.5	0.355	1.185	2.77	2.66	2.21	0.95	99.61	1.60	0.95
11	2′-Deoxyadenosine	Y = 47,300X + 17,500	0.9996	1.289–82.5	0.182	0.608	2.82	3.07	1.98	3.23	100.15	1.97	0.99
12	Isoleucine	Y = 21,100X + 211,000	0.9999	5.098–652.5	1.179	3.929	3.11	3.22	2.15	1.27	99.92	1.20	0.96
13	Inosine	Y = 10,900X − 495	0.9992	5.029–643.8	0.903	3.012	2.63	1.91	1.93	2.07	100.42	2.10	1.00
14	Guanosine	Y = 7560X + 146,000	0.9994	2.495–2555	0.466	1.555	2.72	3.83	1.99	1.45	98.98	1.34	1.01
15	Gallic acid	Y = 4090X + 10,100	0.9999	11.035–2825	3.168	10.560	2.23	2.25	1.38	2.81	99.82	2.18	1.01
16	Leucine	Y = 22,900X + 124,000	0.9994	5.039–645	1.032	3.440	2.76	2.41	1.93	1.73	99.27	1.24	1.04
17	2’-Deoxyguanosine	Y = 14900X − 44000	0.9992	2.698–690.6	0.493	1.645	1.97	2.35	3.87	2.93	100.03	2.87	1.02
18	Phenylalanine	Y = 32,700X + 1,210,000	0.9993	5.137–657.5	0.954	3.181	2.96	2.72	2.35	3.53	99.86	3.27	1.02
19	Protocatechuic acid	Y = 14,000X + 105,000	0.9992	2.808–1437.5	0.665	2.215	1.57	3.38	3.04	2.40	100.81	2.14	0.97
20	Catechin	Y = 968X + 12,800	0.9993	189.453–24,250	40.597	135.324	1.63	2.93	3.29	3.25	100.54	1.63	0.98
21	Chlorogenic acid	Y = 7700X + 35,900	0.9999	2.441–2500	0.631	2.105	3.02	2.15	2.68	3.16	99.67	1.70	1.04
22	Coniferic acid	Y=2490X-39,100	0.9998	4.834–4950	0.780	2.599	2.00	3.27	3.27	2.44	99.79	1.83	0.97
23	Quercetin-3-*O*-(6”-galloyl)-*β*-D-galactopyranoside	Y = 2900X – 122,000	0.9994	9.668–4950	1.040	3.465	3.34	2.70	3.09	2.93	99.83	2.60	0.97
24	Quercetin-3-*O*-(6”-galloyl)-*β*-D-glucopyranoside	Y = 2700X – 97,900	0.9996	9.863–5050	1.885	6.282	1.22	1.01	2.86	2.77	100.10	2.34	0.95
25	Quercetin-3-*O*-*β*-D-glucuronide	Y = 5740X + 238,000	0.9997	61.816–31,650	4.579	15.263	2.75	1.95	2.89	1.85	99.10	1.51	0.97
26	Hyperin	Y = 2350X + 54,100	0.9991	4.824–9880	1.285	4.283	1.27	1.67	1.65	1.37	99.25	2.04	1.03
27	Rutin	Y = 1560X + 5950	0.9998	4.932–1262.5	0.834	2.778	1.26	2.41	4.67	1.16	99.70	1.38	0.98
28	Isoquercitrin	Y = 867X + 461,000	0.9991	24.487–25,075	5.010	16.701	1.39	1.33	2.41	1.75	100.86	1.42	0.97
29	Auicularin	Y = 2800X – 11,100	0.9990	9.766–5000	2.441	8.138	2.79	2.39	1.77	0.86	100.05	2.65	0.96
30	Kaempferol-3,7-bisrhamnoside	Y = 1170X − 1700	0.9998	4.858–2487.5	1.388	4.627	1.26	2.90	3.53	4.45	98.83	1.20	0.94
31	Quercetrin	Y = 3980X + 608,000	0.9997	17.761–36,375	2.264	7.546	1.80	1.77	2.94	1.43	99.97	2.18	0.95
32	Quercetin	Y = 3160X – 212,000	0.9990	9.766–5000	2.307	7.689	3.09	3.86	2.20	1.37	100.58	2.75	0.92
33	Isosakuranetin	Y = 7540X – 11,800	0.9994	3.082–789.1	0.478	1.595	1.12	3.52	3.65	4.58	98.03	2.78	1.03

**Table 3:** Sample information.

Species	No.	Host	Family of Host	Batch No.	Origin
Taxilli Herba	S1	*Morus alba*	Moraceae	2019051901	Wuzhou Guangxi
	S2	*Morus alba*	Moraceae	2019051902	Wuzhou Guangxi
	S3	*Morus alba*	Moraceae	2020121301	Wuzhou Guangxi
	S4	*Morus alba*	Moraceae	2020121302	Wuzhou Guangxi
	S5	*Morus alba*	Moraceae	2020121303	Wuzhou Guangxi
	S6	*Morus alba*	Moraceae	2021021401	Wuzhou Guangxi
	S7	*Morus alba*	Moraceae	2021030101	Wuzhou Guangxi
	S8	*Liquidambar formosana*	Altingiaceae	2019110301	Nanning Guangxi
	S9	*Liquidambar formosana*	Altingiaceae	2019110302	Nanning Guangxi
	S10	*Liquidambar formosana*	Altingiaceae	2020122802	Wuzhou Guangxi
	S11	*Liquidambar formosana*	Altingiaceae	2020122803	Wuzhou Guangxi
	S12	*Cinnamomum camphora*	Lauraceae	2020081801	Nanning Guangxi
	S13	*Cinnamomum camphora*	Lauraceae	2020081802	Nanning Guangxi
	S14	*Clausena lansium*	Rutaceae	2019110302	Nanning Guangxi
	S15	*Clausena lansium*	Rutaceae	2019110303	Nanning Guangxi
	S16	*Clausena lansium*	Rutaceae	2020122812	Wuzhou Guangxi
	S17	*Clausena excavata*	Rutaceae	2019100201	Chongzuo Guangxi
	S18	*Clausena excavata*	Rutaceae	2019100202	Chongzuo Guangxi
	S19	*Tabernaemontana divaricata*	Apocynaceae	2019052005	Wuzhou Guangxi
	S20	*Tabernaemontana divaricata*	Apocynaceae	2019052006	Wuzhou Guangxi
	S21	*Amygdalus persica*	Rosaceae	2020122901	Nanning Guangxi
	S22	*Amygdalus persica*	Rosaceae	2020122902	Nanning Guangxi
	S23	*Amygdalus persica*	Rosaceae	2020122903	Nanning Guangxi
	S24	*Glyptostrobus pensilis*	Cupressaceae	2020120801	Baise Guangxi
	S25	*Glyptostrobus pensilis*	Cupressaceae	2020120802	Baise Guangxi
	S26	*Glyptostrobus pensilis*	Cupressaceae	2020120803	Baise Guangxi
	S27	*Diospyros kaki*	Ebenaceae	2020122816	Wuzhou Guangxi
	S28	*Diospyros kaki*	Ebenaceae	2020122817	Wuzhou Guangxi
	S29	*Ilex latifolia*	Aquifoliaceae	2019051903	Wuzhou Guangxi
	S30	*Ilex latifolia*	Aquifoliaceae	2019051904	Wuzhou Guangxi
	S31	*Crataegus pinnatifida* var. *major*	Rosaceae	2019051906	Wuzhou Guangxi
	S32	*Crataegus pinnatifida* var. *major*	Rosaceae	2019051907	Wuzhou Guangxi
	S33	*Crataegus pinnatifida* var. *major*	Rosaceae	2019051908	Wuzhou Guangxi
	S34	*Passiflora edulis*	Passifloraceae	2019052001	Wuzhou Guangxi
	S35	*Passiflora edulis*	Passifloraceae	2019052002	Wuzhou Guangxi
	S36	*Passiflora edulis*	Passifloraceae	2019052003	Wuzhou Guangxi
	S37	*Pyrus pyrifolia*	Rosaceae	2019051910	Wuzhou Guangxi
	S38	*Pyrus pyrifolia*	Rosaceae	2019051911	Wuzhou Guangxi
Tolypanthi Herba	S39	*Morus alba*	Moraceae	2019070701	Guilin Guangxi
	S40	*Morus alba*	Moraceae	2019070702	Guilin Guangxi
	S41	*Diospyros kaki*	Ebenaceae	2019070706	Guilin Guangxi
	S42	*Morus alba*	Moraceae	2019070703	Guilin Guangxi
	S43	*Morus alba*	Moraceae	2019070704	Guilin Guangxi
	S44	*Diospyros kaki*	Ebenaceae	2019070707	Guilin Guangxi
	S45	*Diospyros kaki*	Ebenaceae	2019070708	Guilin Guangxi

**Table 4:** Quality sequencing of the 45 tested samples.

No.	*r_i_*	Ranking	Difference of (*r_i_*%)	No.	*r_i_*	Ranking	Difference of (*r_i_*%)
S1	0.4537	3	6.2	S24	0.3574	35	26.1
S2	0.4392	4	9.2	S25	0.3564	36	26.3
S3	0.4386	5	9.3	S26	0.3479	39	28.0
S4	0.4308	7	10.9	S27	0.3892	22	19.5
S5	0.4384	6	9.3	S28	0.3831	23	20.8
S6	0.4783	2	1.1	S29	0.3950	17	18.3
S7	0.4836	1	0.0	S30	0.3943	18	18.4
S8	0.3763	26	22.2	S31	0.3916	20	19.0
S9	0.3899	21	19.4	S32	0.3787	25	21.7
S10	0.3816	24	21.1	S33	0.3673	31	24.0
S11	0.3756	27	22.3	S34	0.3748	29	22.5
S12	0.3203	45	33.8	S35	0.3635	33	24.8
S13	0.3276	42	32.3	S36	0.3617	34	25.2
S14	0.3994	15	17.4	S37	0.3686	30	23.8
S15	0.4015	14	17.0	S38	0.3653	32	24.4
S16	0.4071	12	15.8	S39	0.3749	28	22.5
S17	0.3961	16	18.1	S40	0.3378	40	30.1
S18	0.3926	19	18.8	S41	0.3234	43	33.1
S19	0.4096	11	15.3	S42	0.3506	38	27.5
S20	0.4056	13	16.1	S43	0.3555	37	26.5
S21	0.4114	10	14.9	S44	0.3218	44	33.5
S22	0.4298	8	11.1	S45	0.3360	41	30.5
S23	0.4200	9	13.2				

## Data Availability

The data presented in this study are available in Supplementary Materials.

## Supplementary Materials

The following are available online. Figure S1: Effects of methanol concentration, liquid-to-material ratio, and extraction time on extraction yields of quercitrin, Figure S2: Three-dimensional response surface plots showing effects of variables on the extraction yield of quercitrin, Figure S3: Histograms of contents of four kinds of compounds in 45 samples, Table S1: Levels and code of extraction variables used in the Box–Behnken design, Table S2: Box–Behnken experimental design and the results for the extraction yield of quercetrin, Table S3: Analysis of variance of the experimental results of the BBD, Table S4: Contents of 33 analytes in samples.
