# Supplementary material for: Quality Evaluation of Taxilli Herba from Different Hosts Based on Simultaneous Determination of Multiple Bioactive Constituents Combined with Multivariate Statistical Analysis
Source: Molecules. 2021 Dec 10;26(24):7490. doi: 10.3390/molecules26247490 (PMC8703938; doi:10.3390/molecules26247490)
Supplement: Supplementary file 1 [file molecules-26-07490-s001.zip › molecules-1476892-supplementary.pdf]

Quality Evaluation of Taxilli Herba from Different Hosts Based on Simultaneous Determination of Multiple Bioactive Constituents Combined with Multivariate Statistical Analysis

Nan Wu [1], Li Li [2], Zhi-Chen Cai [1], Jia-Huan Yuan [1], Wen-Xin Wang [1], Sheng-Xin Yin [1], Sheng-Jin Liu [1,*], Li-Fang Wei [1], Yu-Qi Mei [1], Cui-Hua Chen [1], Xun-Hong Liu [1,*], Li-Si Zou [1] and Jie Li [1]

Supplementary materials

Supplementary figures

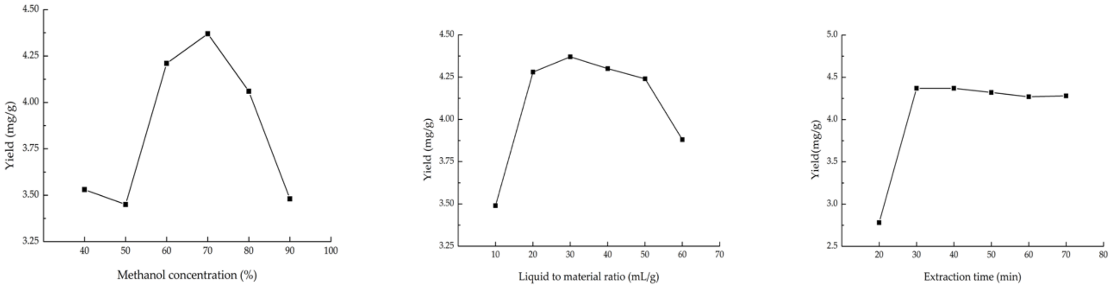

Figure S1. Effects of methanol concentration, liquid to material ratio and extraction time on extraction yields of quercitrin. (“extraction yield (%) = weight of analyte (mg) / weight of dried sample (g) × 100%).

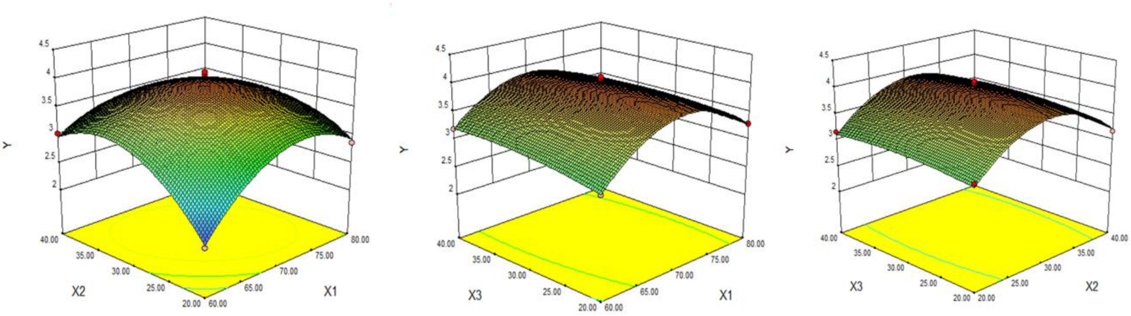

Figure S2. 3D response surface plots showing effects of variables on the extraction yield of quercitrin. X_1. methanol concentration (%), X_2. liquid to material ratio (mL/g) and X_3. extraction time (min).

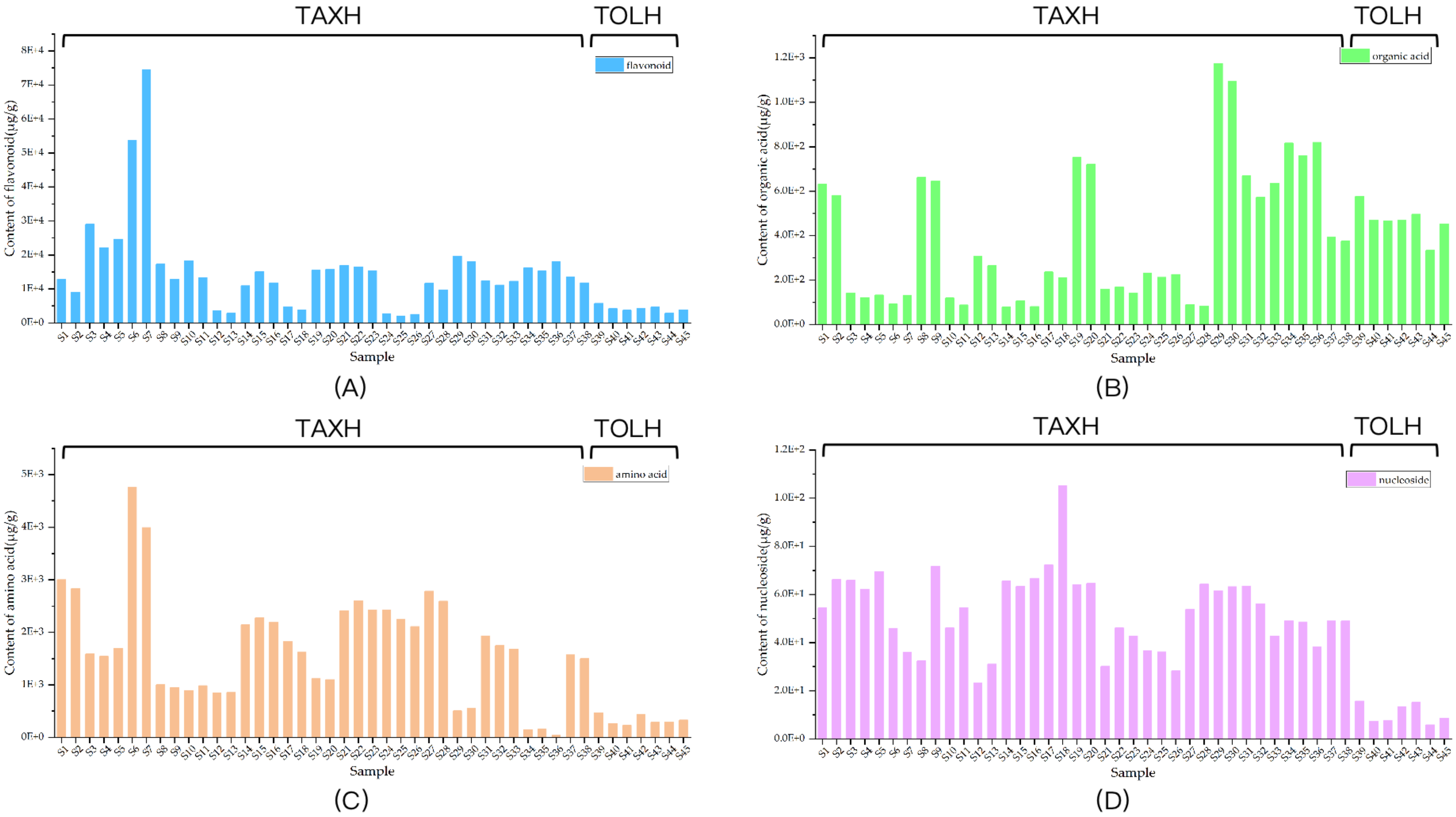

Figure S3. Histograms of contents of four kinds of constituents in 45 samples.

Supplementary tables

Table S1 Levels and code of extraction variables used in Box-Behnken design.

Variable	Symbols Coded	Coded Levels		
		-1	0	1
Methanol concentration (%)	X_1	60	70	80
Liquid to material ratio (mL/g)	X_2	20	30	40
Extraction time (min)	X_3	20	30	40

Table S2 Box-Behnken experimental design and the results for extraction yield of quercetrin.

Run	X_1	X_2	X_3	Response value
	Methanol concentration (%)	Liquid to material ratio (mL/g)	Extraction time (min)	*Y* (mg/g)
1	-1	0	1	3.20
2	-1	-1	0	2.01
3	0	1	-1	3.18
4	1	-1	0	2.86
5	0	0	0	3.82
6	0	-1	1	3.17
7	0	0	0	3.87
8	1	1	0	2.43
9	0	-1	-1	3.10
10	-1	1	0	3.03
11	0	0	0	4.07
12	0	1	1	3.23
13	1	0	1	3.20
14	0	0	0	3.78
15	-1	0	-1	2.95
16	0	0	0	4.11
17	1	0	-1	3.29

Note: "*Y*" the extraction yield of quercetrin. (*Y* (mg/g) = weight of analyte (mg) / weight of dried sample (g) × 100%).

Table S3 Analysis of variance of the experimental results of the BBD.

Parameter	Sum of Squares	Degree of Freedom	Mean Square	*F*-value	*p*-value
Model	4.83	9	0.54	33.39	$<0.0001^{**}$
X_1	0.044	1	0.044	2.72	0.1431
X_2	0.066	1	0.066	4.11	0.0821
X_3	9.940×10-3	1	9.940×10^{-3}	0.62	0.4572

X_1X_2	0.53	1	0.53	32.87	0.0007**
X_1X_3	0.029	1	0.029	1.8	0.2216
X_2X_3	3.600×10-5	1	3.600×10^{-5}	2.242×10^{-3}	0.9636
X_1^2	1.94	1	1.94	120.89	< 0.0001**
X_2^2	1.88	1	1.88	117	< 0.0001**
X_3^2	0.035	1	0.35	2.21	0.181
Residual	0.11	7	0.016		
Lack of Fit	0.025	3	8.369×10^{-3}	0.38	0.7715
Pure Error	0.087	4	0.022		
Cor Total	4.94	16			

Note: $^{**}p \leq 0.01$

Table S4. Contents of 33 constituents in samples. (μg/g, mean ± SD, *n* = 3)

Analyte	S1	S2	S3	S4	S5	S6	S7	S8	S9
Lysine	7.49±0.34	9.38±0.74	7.80±0.38	9.75±0.84	10.83±0.58	153.59±11.20	204.72±1.61	-	-
Histidine	31.32±2.19	20.54±0.60	18.87±0.73	10.01±0.10	12.39±0.62	44.44±0.19	48.92±0.58	4.87±0.19	5.41±0.36
Argnine	163.57±9.56	154.98±10.05	62.34±4.73	40.66±1.28	49.8±3.04	668.28±12.53	524.37±10.68	15.76±1.82	14.85±2.01
Serine	28.75±2.77	14.12±0.14	17.71±1.20	22.4±3.94	35.27±5.73	94.22±6.33	54.79±3.44	22.72±3.04	12.27±3.21
Theronine	47.69±3.60	38.8±1.03	34.74±2.93	29.1±1.93	31.92±3.04	95.96±4.82	80.5±6.40	16.23±2.93	16.59±4.32
Glutamic acid	744.25±5.00	594.28±28.61	563.98±20.83	521.1±13.38	580.35±10.47	797.77±30.54	1383.57±43.22	201.26±30.20	174.66±6.28
Proline	1687.36±30.00	1703.58±10.00	702.6±22.11	688.11±28.73	734.98±21.03	2349.71±79.11	1024.84±32.01	688.41±14.92	643.61±43.46
Valine	21.96±2.06	19.89±0.91	22.21±0.51	21.46±1.92	23.4±1.02	92.79±16.38	82.04±23.99	5.94±1.00	7.91±2.08
Tyrosine	87.41±0.85	92.39±4.54	47.44±1.44	61.43±8.32	77.28±5.44	84.25±9.03	114.84±6.28	12.06±2.47	11.50±0.83
Adenosine	19.04±1.23	29.52±3.34	44.07±2.54	37.61±5.32	39.39±0.27	30.08±8.57	22.84±1.74	25.41±4.28	42.39±3.80
2′-Deoxyadenosine	0.14±0.02	-	0.10±0.01	0.22±0.10	0.16±0.11	0.37±0.03	0.34±0.04	0.06±0.03	0.11±0.03
Isoleucine	80.78±4.04	80.93±4.32	47.78±1.78	59.87±1.29	56.36±5.92	130.76±36.29	177.29±7.50	20.17±4.33	30.70±3.36
Inosine	7.63±0.50	8.97±0.53	3.18±0.19	2.38±0.55	4.99±1.33	3.89±0.03	2.24±0.99	4.98±1.03	6.10±0.94
Guanosine	25.8±3.24	25.86±0.91	16.94±0.45	20.24±3.77	22.93±4.72	10.95±4.88	10.18±2.01	-	21.19±3.67
Gallic acid	448.34±12.38	401.68±15.24	104.38±5.51	91.68±7.00	95.06±3.88	82.06±7.38	121.15±3.12	587.44±45.32	570.73±37.66
Leucine	60.22±3.65	64.17±4.08	41.91±0.90	48.22±5.55	48.27±3.00	157.28±8.33	165.43±9.00	15.65±3.24	24.09±3.82
2'-Deoxyguanosine	1.85±0.20	1.95±0.11	1.64±0.03	1.75±0.87	2.04±0.78	0.48±0.03	0.42±0.04	1.94±0.83	1.91±0.63
Phenylalanine	39.68±0.87	43.24±2.59	20.2±0.12	30.73±4.58	32.24±3.02	92.11±11.47	131.79±5.38	-	8.79±2.11
Protocatechuic acid	41.92±3.87	38.03±1.03	5.78±0.55	6.79±3.22	10.31±4.44	2.75±0.04	2.44±1.00	53.56±3.58	57.53±4.27
Catechin	1490.39±20.28	1184.87±9.66	9242.27±70.95	7250.38±198.04	8028.81±112.29	5296.78±100.47	2840.89±230.11	1643.4±100.00	1401.6±112.32
Chlorogenic acid	134.62±5.13	133.37±0.76	25.25±1.23	16.78±1.03	20.35±3.02	6.82±1.48	5.35±1.22	12.83±4.28	9.11±1.33
Coniferic acid	7.74±0.26	8.11±0.05	6.64±0.11	6.14±0.30	6.85±1.01	0.17±0.05	0.25±0.07	8.86±2.88	8.68±1.03

Quercetin-3-O-(6″-galloyl)-β-D-galactopyranoside	17.52±0.60	17.81±1.03	17.54±2.93	16.09±1.83	17.69±4.20	1.52±0.04	1.00±0.34	19.86±8.76	21.47±4.37
Quercetin-3-O-(6″-galloyl)-β-D-glucopyranoside	15.44±0.37	15.72±2.19	16.01±2.38	13.88±4.22	0.92±0.11	4.74±0.57	4.40±1.22	17.88±2.04	17.66±2.20
Quercetin-3-O-β-D-glucuronide	5354.66±115.19	3128.58±70.00	6639.91±88.37	4374.92±86.20	5306.08±170.62	45786.5±450.37	68355.74±374.56	7373.71±122.48	4813.77±4.58
Hyperin	1089.24±15.91	735.48±36.51	2063.26±36.01	1409.46±99.27	1598.27±86.03	296.32±28.36	377.18±12.33	1745.6±144.89	1307.93±110.31
Rutin	162.99±5.51	128.17±0.89	339.49±15.83	254.38±10.78	262.21±33.18	102.89±10.11	136.51±23.22	188.77±2.60	165.01±12.83
Isoquercitrin	1517.73±22.11	1255.23±11.92	4120.71±128.97	3349.72±70.36	3785.79±102.63	323.44±22.34	366.45±12.83	3158.37±113.73	2809.63±45.33
Auicularin	5.93±0.29	6.71±0.04	6.12±2.10	5.06±1.09	5.24±1.32	0.95±0.40	0.74±0.04	4.10±0.75	3.33±1.20
Kaempferol-3,7-bisrhamnoside	0.59±0.03	0.68±0.02	0.74±0.14	0.56±0.12	0.66±0.12	0.99±0.05	1.16±0.04	0.68±0.32	0.71±0.11
Quercetrin	3183.92±26.51	2605.27±112.03	6567.67±120.38	5387.05±63.00	5560.45±94.77	1798.89±57.90	2239.62±77.34	3133.7±45.46	2318.4±170.32
Quercetin	37.97±0.23	29.68±3.49	32.08±4.82	26.67±1.58	30.3±3.67	48.72±4.33	57.08±7.03	45.86±3.84	42.33±9.22
Isosakuranetin	0.71±0.02	0.60±0.02	0.56±0.18	13.93±1.09	1.11±0.08	0.58±0.11	0.57±0.04	0.61±0.08	0.59±0.37

Note: “-”not detected.

Table S4. Contents of 33 constituents in samples. (continued)

Analyte	S10	S11	S12	S13	S14	S15	S16	S17	S18
Lysine	1.47±0.29	1.83±0.12	-	6.40±0.30	13.71±1.94	12.82±1.21	14.13±3.22	10.10±0.50	9.83±3.21
Histidine	7.53±0.72	3.96±0.13	5.79±0.55	7.02±0.23	6.83±1.22	8.59±1.22	14.64±0.92	20.13±0.87	17.75±1.02
Argnine	29.13±2.83	22.92±1.22	151.60±8.21	138.69±9.79	138.67±2.33	147.99±12.32	144.55±9.33	277.28±8.89	251.31±23.44
Serine	10.12±4.11	10.32±3.42	12.21±3.82	20.40±1.33	30.85±3.61	19.52±0.92	29.79±1.03	43.36±12.07	36.77±2.26
Theronine	32.09±10.11	20.94±5.44	14.89±4.22	13.13±0.38	36.51±3.21	45.02±8.66	35.96±4.20	26.38±2.60	31.86±1.47
Glutamic acid	313.09±11.32	318.71±16.74	243.88±11.92	237.93±47.52	584.50±27.93	625.47±23.25	614.95±45.02	424.14±10.54	370.00±9.76
Proline	390.45±77.22	448.95±34.33	350.01±10.33	345.94±15.82	1058.89±44.22	1163.77±88.89	1055.4±100.48	885.11±60.00	786.58±26.44
Valine	13.74±6.42	13.43±1.83	6.92±2.50	5.21±0.83	24.44±10.22	25.91±8.79	25.65±3.84	12.63±2.30	12.49±3.22

Tyrosine	13.7±2.48	43.87±11.32	34.51±9.30	44.95±3.70	92.35±14.22	70.59±12.07	99.56±13.42	67.02±3.85	61.28±0.23
Adenosine	23.84±8.22	20.07±9.55	9.51±0.27	11.98±3.22	30.41±9.42	33.26±3.08	30.20±4.63	34.79±1.81	68.17±1.23
2′-Deoxyadenosine	0.13±0.06	0.21±0.03	0.06±0.03	0.07±0.01	0.20±0.04	0.13±0.02	0.12±0.05	0.07±0.03	0.09±0.02
Isoleucine	39.25±2.08	43.63±12.11	10.47±3.22	15.27±2.03	73.12±16.03	75.53±6.49	74.91±7.43	21.38±0.94	15.77±0.71
Inosine	3.59±0.92	9.28±2.01	2.16±0.42	3.13±1.03	8.50±0.27	7.40±0.72	9.24±3.02	8.52±1.63	7.68±0.23
Guanosine	16.38±1.84	23.22±3.82	10.16±0.43	14.28±3.88	24.94±3.92	21.49±3.70	25.58±3.53	27.25±7.21	27.41±3.28
Gallic acid	91.87±10.73	68.15±10.29	259.44±34.55	225.4±13.22	65.30±5.55	85.62±8.89	67.38±11.03	176.94±4.00	157.63±13.52
Leucine	34.68±7.88	38.83±5.44	10.01±5.93	15.65±8.03	65.86±10.10	69.63±5.33	68.87±3.42	24.90±1.01	21.10±1.46
2'-Deoxyguanosine	2.16±0.48	1.75±0.72	1.30±0.66	1.59±0.43	1.53±0.77	1.06±0.08	1.58±0.10	1.61±0.74	1.72±0.44
Phenylalanine	5.44±0.88	15.51±3.82	-	3.66±1.02	20.20±3.22	10.34±2.38	19.08±3.53	12.75±1.09	10.31±3.21
Protocatechuic acid	9.84±1.84	7.59±1.00	36.08±3.82	30.57±11.83	2.55±0.72	5.95±0.42	2.81±1.02	44.25±1.66	38.45±4.22
Catechin	4617.72±232.47	3498.8±205.66	965.3±47.67	827.44±17.83	3437.49±163.55	4648.09±105.04	3655.98±89.54	739.31±75.62	649.70±19.48
Chlorogenic acid	9.33±2.77	6.16±7.11	4.13±1.03	2.19±0.37	4.88±1.01	8.38±0.72	4.88±1.03	6.38±0.05	5.57±1.00
Coniferic acid	8.03±4.82	5.88±1.04	6.71±1.03	6.24±0.37	5.58±0.99	6.71±0.72	5.67±2.10	9.24±1.28	8.03±0.73
Quercetin-3-O-(6″-galloyl)-β-D-galactopyranoside	19.91±5.55	13.48±4.32	13.01±4.33	13.16±3.21	13.32±3.82	16.18±3.02	13.80±2.22	15.69±0.73	13.61±3.84
Quercetin-3-O-(6″-galloyl)-β-D-glucopyranoside	18.60±2.05	13.04±5.30	11.32±5.03	11.45±6.16	12.25±3.22	14.47±2.22	12.05±1.03	14.23±1.75	12.11±1.23
Quercetin-3-O-β-D-glucuronide	5300.75±222.47	3730.47±41.63	1225.26±77.28	971.11±11.83	3244.96±71.73	4146.91±39.05	3492.1±45.32	1461.42±19.76	964.53±84.45
Hyperin	745.95±56.04	560.01±68.22	311.99±64.00	230.77±19.65	611.21±36.44	853.40±11.09	638.29±32.11	349.40±5.64	286.60±44.03
Rutin	148.01±49.44	102.34±7.21	46.70±12.50	35.24±9.32	122.17±23.69	176.24±36.05	118.95±14.28	247.58±13.51	200.70±34.22
Isoquercitrin	4119.03±120.77	3284.05±128.97	407.63±9.50	266.36±67.32	1218.13±106.28	1674.41±157.16	1246.33±37.02	1021.61±106.93	812.48±74.02
Auicularin	4.74±2.06	3.15±0.32	1.30±0.70	1.36±0.25	3.00±1.02	3.54±0.44	2.63±1.03	1.46±0.06	1.47±0.74
Kaempferol-3,7-bisrhamnoside	0.78±0.07	0.49±0.06	0.53±18	0.57±0.18	0.53±0.03	0.59±0.06	0.63±0.04	0.62±0.12	0.54±0.26
Quercetrin	3305.89±198.66	2095.29±155.89	594.40±32.53	544.92±34.33	2276.69±72.99	3539.07±57.95	2594.44±145.66	904.03±5.02	888.49±100.48
Quercetin	32.66±4.27	22.15±6.83	22.29±9.32	21.76±6.39	21.79±0.82	27.66±3.85	22.11±5.30	26.07±2.52	21.08±5.39

Isosakuranetin	0.56±0.04	0.91±0.11	0.55±0.03	0.67±0.07	0.67±0.21	0.54±0.10	0.71±0.20	0.60±0.11	0.60±0.03

Table S4. Contents of 33 constituents in samples. (continued)

Analyte	S19	S20	S21	S22	S23	S24	S25	S26	S27
Lysine	1.63±0.32	1.71±0.22	161.79±0.75	75.96±5.32	69.08±3.02	14.09±1.36	13.19±2.72	11.41±5.22	6.14±1.29
Histidine	4.56±1.23	5.28±1.03	41.42±1.63	35.47±8.21	27.10±9.50	9.80±2.01	10.53±1.49	18.15±1.10	8.67±1.10
Argnine	60.89±15.22	57.92±4.38	483.54±41.63	475.74±34.32	465.56±74.52	431.95±52.48	425.44±32.25	377.94±19.51	142.39±12.42
Serine	16.26±5.22	6.30±1.38	26.02±3.38	33.75±12.38	32.28±8.06	18.33±7.90	16.25±7.21	17.66±3.71	20.20±0.70
Theronine	25.89±3.01	12.68±2.32	40.02±7.90	30.69±9.46	29.17±3.38	22.18±1.57	21.60±1.57	33.36±5.17	73.22±1.21
Glutamic acid	161.00±32.94	149.71±2.22	559.73±67.31	612.47±23.74	536.83±18.32	1194.72±486.86	1102.05±110.12	1050.16±90.21	589.47±45.09
Proline	599.51±12.55	610.52±30.63	820.63±74.52	954.04±77.03	937.76±10.58	575.33±74.52	503.57±43.51	481.43±16.37	1751.08±110.29
Valine	15.82±2.26	14.55±1.81	28.82±9.50	34.10±1.32	29.13±0.93	11.81±1.13	12.68±7.55	11.78±3.29	15.34±3.65
Tyrosine	81.60±5.77	91.95±18.80	63.13±1.24	108.87±10.77	80.77±7.36	72.79±37.55	70.56±9.80	53.77±6.26	42.77±5.11
Adenosine	32.98±10.60	32.05±4.80	16.79±1.12	24.28±2.33	23.15±9.61	17.53±4.34	16.73±7.45	13.69±4.66	28.99±6.02
2′-Deoxyadenosine	0.07±0.03	0.04±0.01	0.09±0.03	0.13±0.09	0.12±0.03	0.18±0.04	0.15±0.06	0.12±0.03	0.04±0.02
Isoleucine	49.44±5.69	50.22±4.54	87.90±12.32	115.00±56.20	104.54±32.54	26.08±0.36	23.99±5.82	20.75±1.68	67.11±3.85
Inosine	2.96±1.02	4.59±1.47	1.22±0.03	2.24±0.32	2.68±0.18	2.53±0.93	2.88±0.43	2.48±0.60	7.28±0.87
Guanosine	26.75±3.22	26.56±3.06	10.67±0.93	17.86±1.78	15.09±1.12	14.67±3.79	14.39±1.72	10.42±1.10	15.88±1.10
Gallic acid	612.46±24.80	572.85±23.73	130.58±2.29	142.40±22.04	117.31±8.04	204.78±7.10	181.31±61.55	190.82±14.83	70.11±3.08
Leucine	43.13±4.54	44.37±1.38	87.08±7.79	110.57±19.67	101.01±14.32	34.85±6.32	33.39±8.76	28.56±5.43	61.51±12.07
2'-Deoxyguanosine	1.33±0.32	1.35±0.27	1.30±0.32	1.54±0.47	1.66±0.37	1.76±0.40	2.01±0.05	1.68±0.35	1.69±0.77
Phenylalanine	58.08±3.60	54.55±0.79	8.32±1.13	16.51±0.94	12.60±4.54	12.28±0.38	9.99±0.37	3.12±0.80	3.66±0.38
Protocatechuic acid	116.68±4.29	125.89±1.53	8.25±1.02	8.94±2.49	8.40±1.32	19.56±0.08	24.98±3.55	23.91±1.78	8.03±0.59
Catechin	783.22±28.16	870.00±47.65	7001.57±111.01	6757.64±244.66	6811.49±53.62	486.24±32.24	433.36±16.47	451.88±32.69	4565.18±157.16

Chlorogenic acid	16.25±1.05	16.56±0.94	14.07±2.29	10.14±3.02	10.25±4.43	1.33±0.03	0.65±0.14	4.95±1.27	4.83±1.03
Coniferic acid	5.64±0.14	5.90±0.10	6.16±0.74	6.20±1.77	6.40±1.72	5.74±1.25	5.42±1.33	5.92±1.02	6.81±2.12
Quercetin-3-O-(6″-galloyl)-β-D-galactopyranoside	14.40±1.09	13.94±0.94	14.00±1.21	14.18±2.33	13.58±0.55	12.81±0.69	13.07±0.87	12.83±1.89	16.67±2.77
Quercetin-3-O-(6″-galloyl)-β-D-glucopyranoside	15.48±1.47	14.29±1.09	12.60±1.23	12.79±0.98	12.24±2.29	10.91±3.62	11.15±4.38	11.13±3.21	14.32±1.82
Quercetin-3-O-β-D-glucuronide	4299.41±118.46	4675.71±129.57	3594.55±372.00	2984.72±44.77	2855.76±158.60	1161.32±52.01	819.23±12.57	1091.00±138.27	2910.55±20.28
Hyperin	1346.29±30.55	1282.46±111.83	853.69±79.30	801.82±82.24	804.69±74.25	182.68±3.65	147.68±17.95	185.97±12.42	576.86±32.15
Rutin	220.01±29.43	198.86±4.43	137.15±44.36	138.00±47.25	111.50±7.92	28.17±5.37	29.26±0.87	35.80±10.54	124.5±1.25
Isoquercitrin	5238.41±160.42	4961.59±284.77	2910.27±93.64	3092.09±101.71	2524.60±3.68	192.20±12.60	164.15±10.50	217.78±23.28	981.16±30.00
Auicularin	3.87±0.01	4.19±0.21	2.62±1.22	3.22±0.37	2.45±1.00	1.25±0.43	1.38±0.22	1.43±0.37	3.34±0.43
Kaempferol-3,7-bisrhamnoside	0.53±0.21	0.49±0.07	0.52±0.04	0.59±0.03	0.57±0.22	0.46±0.06	0.52±0.39	0.48±0.06	0.65±0.04
Quercetrin	3609.95±28.47	3738.09±182.50	2366.70±43.22	2594.44±66.39	2176.92±100.32	487.99±17.13	434.67±60.34	506.23±47.47	2429.54±115.67
Quercetin	35.70±1.31	37.69±0.23	24.26±1.18	24.07±7.66	22.10±1.49	20.61±2.28	20.82±3.61	22.17±2.10	27.07±2.60
Isosakuranetin	0.64±0.40	0.59±0.02	0.53±0.04	0.76±0.08	7.95±1.32	0.91±0.11	0.98±0.19	0.53±0.03	0.52±0.04

Table S4. Contents of 33 constituents in samples. (continued)

Analyte	S28	S29	S30	S31	S32	S33	S34	S35	S36
Lysine	6.36±0.78	1.67±0.76	-	0.22±0.02	-	0.18±0.01	-	-	-
Histidine	5.43±0.55	3.93±1.75	4.03±0.23	3.72±0.33	3.74±0.36	5.01±0.42	4.71±0.44	4.62±0.47	3.72±0.35
Argnine	132.50±4.58	15.87±4.45	14.28±1.27	34.93±1.56	31.65±1.23	9.25±1.04	10.05±1.09	7.22±0.51	8.61±0.41
Serine	21.53±3.34	3.78±0.10	18.92±2.88	5.91±1.11	13.16±0.89	0.22±0.01	12.51±0.92	21.22±1.11	-
Theronine	36.52±1.85	4.07±0.62	4.95±1.04	13.53±2.03	15.74±0.68	23.00±0.95	-	-	24.70±1.09
Glutamic acid	558.49±20.27	47.28±1.26	52.66±0.74	318.28±8.59	273.42±5.17	276.60±8.37	106.36±3.68	102.71±2.98	-

Proline	1600.69±52.50	384.52±4.59	372.86±12.14	1309.23±43.39	1179.82±69.05	1187.08±7.11	-	-	-
Valine	14.29±0.67	5.32±0.58	5.37±0.78	17.01±1.53	16.08±1.99	15.38±0.88	2.38±0.03	2.90±0.18	6.31±0.74
Tyrosine	57.14±3.80	6.91±0.21	31.37±7.00	85.17±3.29	82.11±4.16	54.09±1.92	-	-	-
Adenosine	33.95±16.88	34.84±1.27	34.66±3.05	32.16±1.06	27.37±1.10	25.71±0.95	28.12±1.05	29.09±2.56	25.11±0.96
2′-Deoxyadenosine	0.11±0.03	0.12±0.01	0.09±0.01	0.05±0.01	0.01±0.00	0.09±0.01	0.03±0.01	0.15±0.02	0.07±0.01
Isoleucine	76.06±3.36	15.25±1.18	18.91±0.58	67.46±2.11	59.48±2.17	54.79±1.02	5.80±0.09	7.93±0.10	-
Inosine	8.44±0.83	4.02±1.05	3.46±0.36	7.78±0.52	8.22±0.37	4.12±0.03	3.40±0.06	3.33±0.08	1.92±0.01
Guanosine	20.24±0.21	21.02±0.57	23.35±1.96	21.73±0.85	19.00±2.10	11.47±0.35	15.59±0.86	14.39±0.62	9.90±0.17
Gallic acid	68.49±6.12	1026.56±35.01	965.16±54.10	560.72±5.79	493.93±17.06	536.72±4.09	638.74±26.09	591.07±12.07	648.92±4.55
Leucine	69.49±1.01	11.58±0.60	17.52±3.08	52.05±2.17	46.80±1.74	44.85±0.75	6.38±0.07	7.79±0.66	-
2'-Deoxyguanosine	1.49±0.17	1.47±0.13	1.66±0.16	1.82±0.15	1.57±0.06	1.26±0.09	1.90±0.11	1.60±0.09	1.34±0.38
Phenylalanine	12.91±0.95	6.05±0.06	16.43±0.33	24.88±0.95	22.73±0.44	12.80±0.95	1.88±0.09	0.37±0.02	-
Protocatechuic acid	5.85±0.36	96.79±5.55	85.48±0.29	98.74±1.78	70.22±3.17	86.98±3.19	168.90±5.00	163.15±6.99	162.02±3.67
Catechin	3993.17±118.43	1155.59±16.04	1014.35±58.28	529.41±2.07	448.78±4.98	538.93±7.06	1367.60±26.01	1012.36±36.08	1522.04±17.88
Chlorogenic acid	2.61±0.12	44.59±4.43	37.57±1.93	3.98±0.06	3.90±0.02	6.44±0.07	0.77±0.01	0.53±0.06	2.58±0.51
Coniferic acid	5.53±1.33	6.06±1.75	6.48±0.77	5.57±0.09	5.46±0.09	5.61±0.05	7.75±0.09	5.86±0.25	5.72±0.44
Quercetin-3-O-(6″-galloyl)-β-D-galactopyranoside	13.51±0.96	14.74±4.80	15.92±2.38	12.96±0.16	13.49±1.17	13.13±0.15	19.12±1.06	14.07±0.85	14.12±0.25
Quercetin-3-O-(6″-galloyl)-β-D-glucopyranoside	11.68±1.73	16.00±5.46	16.29±0.24	11.76±0.78	11.83±0.19	12.09±0.51	18.04±1.07	13.90±1.09	14.34±044
Quercetin-3-O-β-D-glucuronide	2271.08±103.25	5425.27±52.20	5276.65±61.53	5635.54±79.04	5269.47±69.07	5376.21±63.01	4577.67±56.22	4711.36±51.95	5270.52±58.09
Hyperin	418.12±10.21	1346.82±56.20	1238.79±27.22	593.45±4.68	590.89±4.88	655.03±9.74	992.18±7.09	1015.44±10.88	1237.49±20.09
Rutin	101.14±20.04	214.33±12.58	161.8±6.97	142.6±3.27	125.28±3.68	155.74±5.68	210.22±5.96	185.36±6.07	243.28±10.99
Isoquercitrin	767.67±51.25	5794.36±154.32	4910.50±32.16	1865.83±69.57	1516.13±56.14	1967.02±32.09	4291.14±79.04	4234.10±69.07	5207.97±67.09
Auicularin	2.02±0.55	4.05±0.56	3.38±0.67	3.68±0.21	3.24±0.17	4.32±0.01	5.37±0.47	2.94±0.11	3.36±0.37
Kaempferol-3,7-bisrhamnoside	0.49±0.11	0.48±0.02	0.59±0.01	0.46±0.03	0.53±0.05	0.54±0.01	0.72±0.05	0.47±0.03	0.48±0.01
Quercetrin	2101.99±168.74	5609.68±30.57	5282.62±81.35	3597.03±79.99	3129.42±68.93	3500.38±58.05	4678.81±85.98	4038.79±66.40	4518.09±61.00

Quercetin	21.70±5.00	82.44±1.01	57.97±8.32	37.33±1.17	36.48±1.55	41.62±1.79	41.93±1.44	37.49±2.06	45.51±1.89
Isosakuranetin	1.36±0.13	0.57±0.07	0.63±0.22	0.67±0.06	0.70±0.02	0.59±0.02	0.58±0.09	0.73±0.11	0.62±0.11

Table S4. Contents of 33 constituents in samples. (continued)

Analyte	S37	S38	S39	S40	S41	S42	S43	S44	S45
Lysine	0.36±0.04	0.23±0.03	4.17±0.21	2.20±0.11	1.49±0.18	9.86±0.45	10.78±0.41	2.56±0.33	4.17±0.38
Histidine	7.93±0.52	3.79±0.23	6.50±0.12	6.52±0.42	9.50±0.51	-	2.51±0.26	8.58±0.51	-
Argnine	97.21±30.78	94.89±25.19	73.09±28.22	19.42±3.10	13.89±1.11	64.59±2.73	70.24±5.67	11.88±0.98	18.17±0.68
Serine	2.73±0.08	12.66±0.82	15.99±1.03	8.81±0.66	-	35.19±1.89	7.22±0.71	16.45±1.09	22.22±0.77
Theronine	13.38±1.05	11.12±1.11	24.53±0.77	53.30±2.35	29.70±1.27	61.12±2.10	54.19±1.26	61.50±1.08	70.44±1.75
Glutamic acid	505.03±13.09	485.17±7.83	156.35±4.38	79.28±1.73	97.92±3.84	121.25±5.49	8.28±0.87	78.94±1.22	116.16±5.61
Proline	854.69±13.66	799.58±10.95	120.39±3.55	49.32±1.83	47.52±3.99	85.95±2.77	90.33±2.71	38.13±1.81	56.84±3.99
Valine	3.70±0.65	4.62±0.56	13.88±0.89	11.48±0.81	1.55±0.10	21.04±1.09	10.07±0.91	61.44±4.02	18.76±1.89
Tyrosine	28.72±1.44	33.81±1.09	20.58±1.24	12.56±0.95	13.32±0.66	5.42±0.71	5.15±0.25	2.34±0.04	3.68±0.06
Adenosine	23.64±1.25	22.85±0.95	7.64±0.42	3.57±0.11	3.82±0.97	6.33±0.22	7.54±0.76	2.58±0.25	3.82±0.11
2′-Deoxyadenosine	0.07±0.01	0.10±0.01	0.17±0.01	0.18±0.01	0.15±0.01	0.24±0.01	0.26±0.01	0.24±0.03	0.22±0.01
Isoleucine	22.37±1.89	23.99±1.27	5.99±0.62	1.22±0.07	0.65±0.03	5.95±0.29	4.91±0.72	0.57±0.05	0.78±0.07
Inosine	4.77±0.97	4.78±0.45	0.86±0.06	0.42±0.07	0.71±0.02	0.53±0.06	0.87±0.09	0.35±0.01	1.03±0.11
Guanosine	18.82±2.11	19.87±1.88	6.74±0.61	2.90±0.27	2.88±0.12	5.90±0.67	6.18±0.10	2.44±0.11	3.12±0.15
Gallic acid	251.48±9.05	245.86±6.73	482.66±8.82	389.46±7.11	385.23±3.85	392.33±8.63	409.60±7.09	269.11±6.03	365.55±6.91
Leucine	19.11±1.68	18.31±0.89	4.52±0.42	-	-	8.06±0.26	3.75±0.41	0.04±0.01	2.27±0.79
2'-Deoxyguanosine	1.62±0.08	1.37±0.21	0.28±0.06	0.25±0.03	0.17±0.03	0.25±0.02	0.37±0.06	0.24±0.01	0.40±0.02
Phenylalanine	12.88±0.99	15.26±0.66	25.88±1.09	20.02±0.85	12.87±0.81	24.14±1.66	25.40±2.89	12.12±0.61	16.18±0.24
Protocatechuic acid	128.41±3.11	116.53±2.88	64.42±1.22	65.65±1.56	68.96±2.72	46.26±4.69	50.49±3.55	46.95±1.99	63.17±2.88

Catechin	281.97±4.69	205.35±6.04	1202.27±15.84	687.87±9.07	705.13±19.61	943.83±12.94	985.78±40.17	502.92±8.05	703.31±15.05
Chlorogenic acid	7.99±0.13	6.83±0.63	26.86±1.99	11.21±0.67	10.47±0.96	19.12±1.16	22.09±3.01	6.51±0.46	10.02±0.91
Coniferic acid	5.62±0.18	5.67±0.88	2.32±0.86	2.88±0.80	2.23±0.08	12.36±0.95	13.29±1.89	10.87±0.16	13.36±0.56
Quercetin-3-O-(6″-galloyl)-β-D-galactopyranoside	13.15±0.23	12.88±1.00	3.81±0.11	7.89±0.22	3.57±0.21	9.04±1.02	10.89±0.97	7.86±0.24	9.71±0.44
Quercetin-3-O-(6″-galloyl)-β-D-glucopyranoside	11.96±0.97	11.93±0.95	126.00±2.35	111.19±2.65	123.57±5.19	87.12±1.55	96.49±4.22	82.97±2.01	107.66±5.91
Quercetin-3-O-β-D-glucuronide	5629.91±44.63	4824.95±69.41	130.97±2.11	205.41±1.98	79.61±4.77	161.75±3.68	177.32±7.83	123.67±2.96	203.28±7.88
Hyperin	682.18±8.82	657.32±7.90	2.99±0.04	0.11±0.16	0.41±0.02	4.44±0.61	4.06±0.71	1.43±0.08	2.82±0.11
Rutin	157.45±6.17	168.19±3.99	30.50±0.99	6.95±0.17	3.94±0.24	25.63±4.80	27.88±0.55	5.45±0.02	7.47±0.72
Isoquercitrin	2642.20±35.79	2558.28±24.51	1029.48±2.33	695.02±2.16	674.01±14.07	769.66±19.66	846.29±21.63	508.04±8.96	657.03±9.06
Auicularin	3.19±0.11	3.66±0.74	113.04±1.85	18.80±0.79	13.55±0.76	75.13±2.87	85.63±7.55	12.15±0.71	17.02±0.96
Kaempferol-3,7-bisrhamnoside	0.55±0.02	0.51±0.07	2.25±0.07	2.44±0.24	2.25±0.28	0.16±0.01	0.24±0.01	0.14±0.01	0.20±0.09
Quercetrin	3985.24±49.63	3348.14±41.01	2118.16±45.89	2221.8±18.93	1898.34±36.94	1721.49±44.81	1867.1±44.10	1588.1±51.00	2076.94±49.06
Quercetin	34.07±1.56	30.30±1.07	993.77±30.78	256.03±4.96	244.78±3.97	541.10±5.93	617.07±8.09	44.25±1.96	57.75±6.77
Isosakuranetin	0.76±0.05	0.73±0.04	0.70±0.06	2.14±0.08	0.57±0.88	0.56±0.06	0.73±0.07	0.40±0.04	0.52±0.60
